# Retrospective Analysis of *Clostridioides difficile* Infection Rates and Outcomes in Hospitalized Patients during the COVID-19 Pandemic: A Unicenter Study in Reus, Spain

**DOI:** 10.3390/jcm13102799

**Published:** 2024-05-09

**Authors:** Simona Iftimie, Ana F. López-Azcona, Mireia Corchero-Valverde, Antonio Peralta-Vázquez, Laia Revuelta López-Cordón, Carles García-Cervera, Luís Manuel Fernández-Domínguez, Jordi Camps, Jorge Joven, Antoni Castro

**Affiliations:** 1 Department of Internal Medicine, Institut d’Investigació Sanitària Pere Virgili, Hospital Universitari de Sant Joan, Universitat Rovira i Virgili, 43204 Reus, Spain; simona.mihaela@salutsantjoan.cat (S.I.); anafelisa.lopez@salutsantjoan.cat (A.F.L.-A.); mireiacorchero@gmail.com (M.C.-V.); antoniopervaz@gmail.com (A.P.-V.); laiarevueltalo@gmail.com (L.R.L.-C.); carles.garcia@salutsantjoan.cat (C.G.-C.); antoni.castro@urv.cat (A.C.); 2 Laboratori de Referència Camp de Tarragona i Terres de l’Ebre, Hospital Universitari de Sant Joan, 43204 Reus, Spain; luismanuel.fernandez@salutsantjoan.cat; 3 Unitat de Recerca Biomèdica, Institut d’Investigació Sanitària Pere Virgili, Hospital Universitari de Sant Joan, Universitat Rovira i Virgili, 43204 Reus, Spain; jorge.joven@salutsantjoan.cat

**Keywords:** cancer, clostridioides difficile, COVID-19, infectious diseases, pandemic, risk factors

## Abstract

**Background:** *Clostridioides difficile* infections (CDI) vary in severity from mild diarrhea to life-threatening conditions like pseudomembranous colitis or toxic megacolon, often leading to sepsis and death. The COVID-19 pandemic prompted changes in healthcare practices, potentially affecting CDI incidence, though reported data are inconclusive. We studied factors influencing CDI incidence and outcomes at a university hospital throughout the COVID-19 pandemic years. **Methods:** We conducted a retrospective study on all adult hospitalized CDI cases from 1 January 2020 to 31 December 2022 in Hospital Universitari de Sant Joan in Reus. We collected demographic information, comorbid conditions, and concurrent infections. **Results:** While overall CDI and COVID-19 rates decreased in 2022, a notable increase in CDI infections was observed among oncological patients and those undergoing some aggressive treatments, such as colonoscopies or gastroscopies. The prevalence of comorbidities remained unmodified, and there were declines in prior gastrointestinal surgeries and proton pump inhibitor prescriptions. Factors associated with patient fatality or prolonged hospitalization included older age, cancer, chronic kidney disease, higher Charlson and McCabe indices, elevated C-reactive protein, and low albumin concentrations. **Conclusions:** Our study shows the evolving landscape of CDI during the COVID-19 pandemic and emphasizes the impact of delayed diagnoses and treatments exacerbated by telemedicine adoption. Identified risk factors for CDI-related mortality or prolonged hospital stays underscore the importance of targeted interventions in high-risk populations.

## 1. Introduction

*Clostridioides difficile* is an anaerobic, Gram-positive, spore-forming bacillus that can proliferate in the intestinal lumen and stands as the primary etiological agent of nosocomial diarrhea [1,2]. The pathogenic spectrum of this microorganism gives rise to a variety of illnesses collectively termed *C. difficile* infections (CDI), ranging from uncomplicated diarrhea to severe conditions like pseudomembranous colitis and toxic megacolon, with potential outcomes including sepsis and fatality [3]. CDI represents an enduring and significant global public health concern, typically arising after disturbances in the normal gut microbiota caused by antibiotic usage [4]. Recent reports have documented an upsurge in CDI in Spain and other Western countries, attributed to heightened clinical suspicion and enhanced diagnostic sensitivity [5]. According to the VINCat registry (a program of the Health Service of the Autonomous Community of Catalonia that establishes a unified surveillance system for nosocomial infections), the incidence rate increased from 2.20 cases per 10,000 hospital stays in 2011 to 3.41 in 2016 [6,7]. This escalation held statistical significance across all CDI categories, including nosocomial, healthcare-related, and community-acquired. Furthermore, there has been a noteworthy surge in the rate of hospitalizations attributed to CDI, escalating from 3.9 cases per 100,000 persons in 2003 to 12.9 in 2013–2015 [7]. The main risk factors for CDI are antibiotic use, advanced age, environmental contamination, and comorbidities such as gastrointestinal diseases or immunodeficiency [8,9]. 

The coronavirus disease 2019 (COVID-19) outbreak prompted an extensive reorganization of healthcare services worldwide, with a significant dependence on robust infection prevention and control measures, including stringent adherence to hand hygiene and proper utilization of personal protective equipment. Theoretically, this heightened emphasis on prevention practices may have decreased the incidence of CDI and other hospital-acquired infections. Conversely, increased use of antibiotics to treat pneumonia and respiratory conditions associated with the virus may have produced the opposite effect [6,10,11,12]. The reported findings are inconclusive, with the majority indicating either no impact or a decrease in CDI rates during the initial wave of COVID-19 [13,14,15,16,17,18,19]. Nevertheless, as the pandemic evolved, so too could its impact on CDI incidence. The clinical characteristics of patients with COVID-19 and the treatments received have undergone enormous changes in recent years. While the initial wave of the pandemic witnessed stringent closures, restricted hospital activities, and a notable lack of population protection, recent times have seen a widespread implementation of effective vaccines, well-established medical protocols, more effective treatments, and shorter hospital stays [20,21,22]. These advancements make it probable that the impact on the incidence of CDI will vary across different pandemic years. As such, we conducted a study to examine the factors influencing the incidence and outcomes of CDI within a university hospital in Reus, Spain, throughout the COVID-19 pandemic years.

## 2. Materials and Methods

We conducted a retrospective study on all hospitalized CDI cases in our hospital from 1 January 2020 to 31 December 2022. The facility, belonging to the Hospital Network for Public Use in the Autonomous Community of Catalonia, Spain, accommodates 367 beds dedicated to hospitalization and an Intensive Care Unit with 20 beds. As a general hospital, it serves a population exceeding 175,000 inhabitants, encompassing primary care facilities and elderly residences in the region. Additionally, the hospital assumes the role of a referral center for the disciplines of Oncology and Radiotherapy, catering to the entirety of the Tarragona province, with approximately 550,000 inhabitants. 

The sole inclusion criterion was being a hospitalized patient aged 18 years or older, treated in any hospital department, and meeting the CDI case definition specified below. We excluded asymptomatic patients, even if they were carriers of a toxin-producing strain. We also excluded patients with a previous history of CDI or those admitted to palliative care units. The inclusion and exclusion criteria were defined by VINCat and were common to all public hospitals in the Autonomous Community of Catalonia, thus facilitating the collection and comparison of results [23]. We documented demographic information, comorbid conditions, and concurrent acute or chronic infections. The research staff manually collected the clinical and demographic data from the computerized medical records, with team members reviewing the documents individually. The McCabe score, which indicates clinical prognosis [24], and the Charlson index, utilized for categorizing patient comorbidities [25], were recorded.

In this report, we use the following definitions:

CDI case: Patient with diarrhea, defined as >3 unformed stools in 24 consecutive hours or less, or toxic megacolon with no other known cause, who has (1) a positive laboratory result for toxin A or B in stool samples or isolation of a toxin-producing strain in stools or detection by molecular techniques of a toxin-producing strain; and (2) an endoscopic, surgical or histological examination that confirms the diagnosis of pseudomembranous colitis.

Nosocomial CDI: CDI identified >48 h after admission and before discharge.

CDI associated with the health system: CDI beginning in the community or the first 48 h from admission, identified in patients who have been discharged from a health center (hospital, residence, or social health center) ≤4 weeks before the onset of symptoms.

Community-acquired CDI: CDI that begins in the community or within the first 48 h of admission, identified in patients with no history of admission to a healthcare facility or who have been discharged >4 weeks before the onset of symptoms.

CDI diagnosis followed the algorithm endorsed by the European Society of Clinical Microbiology and Infectious Diseases [26]. CDI was confirmed with a positive result for both the immunochromatographic detections of glutamate dehydrogenase and toxins A/B (MonlabTest^®^, Monlab S.L., Cornellà, Spain). Additionally, CDI diagnosis was confirmed in cases where one of the previous results was negative, but a positive result emerged through molecular detection methods. SARS-CoV-2 infection was confirmed by an antigen test or reverse transcription-polymerase chain reaction, as previously reported [27].

Data are shown as medians and interquartile ranges or as numbers and percentages. Statistical comparisons between any two groups were performed with the Mann–Whitney U test (quantitative variables) or the χ^2^ test (categorical variables). Statistical significance was set at *p* ≤ 0.05. All calculations were made using the SPSS 25.0 statistical package (SPSS Inc., Chicago, IL, USA).

## 3. Results

### 3.1. Differential Clinical Characteristics of CDI across Three Years of the COVID-19 Pandemic

Table 1 shows the ratio of patients admitted for CDI or COVID-19 in our hospital on the total number of admissions and stays, broken down according to the three years of study. A decrease in the incidence of CDI and COVID-19 per 1000 admissions was observed in 2022 compared to previous years. The clinical characteristics of the patients are shown in Table 2. Age and sex distribution did not show any major variations. In 2022, the number of patients admitted to the Oncology department significantly rose. Some patients experienced remarkably longer stays in 2021 compared to 2020 or 2022, although the differences did not reach statistical significance. No substantial changes were identified in the prevalence of comorbidities. The number of patients with a history of gastrointestinal surgery and those with prescribed proton pump inhibitors declined. Conversely, the number of patients treated with colon or gastroscopy procedures increased in 2021 and stabilized in 2022. In oncological patients, no changes were observed in the type of cancer, its extent, or the therapeutic interventions applied. The ratio of patients with a Charlson index >5 was lower in 2021, and there were no differences in the McCabe index, laboratory results, recurrence rates, or mortality between the observed periods.

A total of 58 patients were prescribed a single antibiotic, while 68 received a combination of two or more. The predominant antibiotics administered were cephalosporins (49 cases), penicillins and their derivatives (46 cases), monobactams (30 cases), quinolones (25 cases), and linezolid (23 cases). Separating the cases by year, we found 47 patients treated with antibiotics in 2020 (62.6%), 42 in 2021 (64.6%), and 37 in 2022 (53.6). This decrease in the last year coincided with a lower incidence of CDI per 1000 admissions (Table 1).

### 3.2. Factors Related to Patient Fatality and Length of Hospital Stay

In Table 3, the characteristics of 27 deceased patients are compared to those of the survivors. The deceased individuals, on average, were older and showed a markedly higher frequency of admissions to the Oncology department. Regarding their comorbidities, they were more likely to suffer from chronic kidney disease or cancer than the survivors. Among the deceased patients with cancer, there was a notable prevalence of lung cancer or metastasis. Deceased patients had a higher frequency of prior hospital admissions. The Charlson and McCabe indices were consistently higher in this group. Additionally, elevated leukocyte and C-reactive protein concentrations were observed, while the albumin concentration was lower than in survivors.

Table 4 shows the characteristics of patients necessitating prolonged hospitalization, defined arbitrarily with a cut-off point set at five days, compared to those with shorter stays. Individuals requiring extended hospitalization were more frequently admitted to the Oncology department and exhibited a higher prevalence of diabetes mellitus or cancer. Among the subset of cancer patients, a higher occurrence of metastases was observed, coupled with a more frequent history of radiation therapy. The extended-stay patients consistently manifested elevated Charlson and McCabe indices, heightened serum C-reactive protein concentrations, and lower albumin concentrations than those with shorter stays.

## 4. Discussion

This study identified distinct clinical characteristics among CDI patients throughout different phases of the COVID-19 outbreak. Hospital clinical practices have significantly differed over the three years of the pandemic. During the onset of the COVID-19 pandemic, patients presenting with infection manifested severe clinical symptoms. Without tailored antiviral therapeutics, the approach primarily revolved around addressing bacterial pneumonia-like manifestations. Due to the surge in patient admissions, many hospitals adopted a quasi-monolithic focus on managing COVID-19 cases, thereby administering antibacterial agents alongside stringent isolation protocols. In 2020, there were widespread home confinements, postponed doctor visits, a surge in telemedicine usage, delays in diagnostics, a deficiency in effective anti-COVID-19 therapies, evident confusion within hospital and social environments, and a predominant focus on COVID-19 and the prevention of its nosocomial transmission. Transition characterized 2021, marked by the global rollout of effective vaccines, enhanced understanding and confidence regarding pandemic characteristics, and a gradual return to normal hospital activities. By the second half of 2022, the situation had essentially normalized, with a large portion of the population vaccinated and hospitals and healthcare resembling pre-pandemic times [28,29,30]. Notably, infection prevention guidelines underwent substantial changes, incorporating universal masking. These alterations in routine clinical practice have necessarily influenced CDI features. 

In 2022, we observed a decrease in the incidence of CDI that mirrored the decline in COVID-19 cases. We attribute this trend to the gradual normalization of clinical activities, which facilitated increased attention to other clinical scenarios alongside a reduction in severe COVID-19 cases. To our knowledge, these findings have not been previously reported. Previous studies aimed to elucidate the relationship between CDI and COVID-19, but variations in methodologies have resulted in inconclusive outcomes. Allegretti et al. [31] reported no increase in CDI rates among COVID-19 patients compared to non-COVID-19 cases. Similarly, Luo et al. [13] and Sinnathamby et al. [32] found no significant difference in CDI rates between the pre-pandemic and pandemic periods. Conversely, several authors [16,33,34] noted a reduction in CDI rates during the early stages of the outbreak compared to the pre-pandemic period. Most studies suggest no discernible increase in CDI during the initial phases of the pandemic [35]. However, our investigation reveals that CDI incidence declined alongside COVID-19 cases in the latter stages.

A striking trend within our cohort is the notable surge in infected oncological patients and those undergoing aggressive treatments, such as gastroscopies or colonoscopies, in 2022 compared to previous years. In the initial two years of the pandemic, we witnessed a widespread transition to telemedicine and public advisories urging individuals to seek in-person medical care only when absolutely necessary. This precautionary stance led to delayed diagnoses and treatments for numerous conditions, including cancer [36]. In the United States, substantial declines have been documented across virtually all non-COVID-19-related healthcare interactions, encompassing emergency department visits [37], outpatient hospital visits [38], surgeries [39], and even myocardial infarctions [40]. Similarly, in Spain [41] and within our hospital, there was a notable decrease in non-urgent diagnoses and treatments during the pandemic’s early years. The gradual return to standard medical practices likely explains the uptick in Oncology patients and individuals undergoing procedures such as gastroscopies or colonoscopies. 

We did not observe any major differences in the patients’ comorbidities, whether the CDI was acquired within the hospital, another social health center, or the community;in the majority of previous treatments and illnesses; as well as in the severity of their diseases measured by the Charlson and McCabe indices. However, some noteworthy trends are worth discussing despite failing to reach statistical significance. For instance, in 2022, a higher number of patients with previous admissions stood out despite receiving less antibiotic therapy. These data raise the question of whether there is horizontal transmission of spores during these admissions, indicating possible infection outbreaks [40]. Unfortunately, we were unable to pinpoint the cases in time and space. Another significant issue is the wide range of post-CDI admission days, particularly notable in 2021, where some patients had to be admitted for many weeks. This observation reflects the inherent shortcomings of telemedicine during 2020 and 2021 and the inevitable reality that hospital care was largely redirected towards COVID-19 cases.

It is noteworthy that the majority of CDI cases originate from the community and hospitals. This underscores the significance of adopting antimicrobial stewardship programs globally, as advocated by the World Health Organization [42]. Such programs aim to implement evidence-based guidelines for prescribing and administering antimicrobials, thereby mitigating drug misuse.

Our subsequent aim was to investigate the factors influencing mortality or extended hospital stays post-CDI. The factors were similar across both scenarios. These individuals were characterized by advanced age compared to those who survived, along with a higher prevalence of chronic kidney disease or cancer as concurrent ailments. Lung cancer or metastatic cancer featured prominently among their comorbidities. Consequently, they exhibited a more frequent history of immunosuppressive treatment and recent hospitalization within three months preceding the CDI diagnosis. The Charlson and McCabe indices indicated a more severe disease prognosis, while leukocyte count, albumin levels, and C-reactive protein were more altered. These findings underscore the significance of these parameters as potential indicators of heightened mortality risk or prolonged hospitalization in this patient population. Our findings conform to the existing literature highlighting numerous risk factors associated with mortality from CDI, encompassing cancer, chronic kidney, cardiovascular, or liver diseases [43,44,45,46]. These comorbidities significantly influence the Charlson index, and it has been suggested that tailoring antibiotic treatment according to Charlson index severity may yield superior efficacy compared to strategies based on laboratory findings [47].

The present study may be limited by its sample size and the demographics of the population studied. The cohort size and composition may only partially represent the broader population affected by CDI, limiting the generalizability of the findings. In addition, the retrospective nature of this study based on the review of clinical databases is not conducive to precise research outcomes since, inevitably, some data may not have been retrieved that could have the potential to impact the outcomes observed. Moreover, being a single-center study in a medium-sized hospital, the cohort size may not be sufficiently robust to explore certain intriguing facets. Expressly, the potential correlations between individual antibiotic usage and CDI incidence warrant deeper scrutiny, yet the limited scope of our current dataset may preclude exhaustive analysis in this regard. 

In conclusion, our study sheds light on the evolving landscape of CDI amidst the COVID-19 pandemic in a medium-sized public hospital in Western Europe. The shifts in clinical practices and healthcare utilization in 2022 were associated with decreased CDI and COVID-19 incidences. However, a striking trend emerged with a significant increase in CDI cases among oncological patients and those undergoing aggressive treatments, likely reflecting delayed diagnoses and treatments during the pandemic’s earlier stages, exacerbated by the widespread adoption of telemedicine. The persistently low co-infection rates of C. difficile and COVID-19, alongside consistent patient comorbidities and disease severity indices, underscore the resilience of CDI dynamics amidst pandemic disruptions. Notably, advanced age, chronic kidney disease, and cancer emerged as key risk factors for mortality or prolonged hospital stays post-CDI, echoing the existing literature.

As we navigate the aftermath of the pandemic, it is imperative to address the challenges posed by telemedicine in facilitating timely diagnoses and treatments, particularly for vulnerable populations. Targeted interventions and healthcare policies should prioritize early detection and management of CDI, especially among high-risk individuals, while considering tailored antibiotic treatments guided by comprehensive risk assessment tools such as the Charlson index. In this sense, creating profiles based on history, clinical, and analytical data can help identify patients with a higher mortality risk. Prioritizing treatments with very effective yet expensive drugs, such as fidaxomicin or bezlotoxumab, for these high-risk patients could significantly improve outcomes [5]. Additionally, our results highlight the importance of managing easily controllable risk factors, such as treatment with antibiotics or proton pump inhibitors and minimizing the length of hospital stays. An important lesson that can be drawn from this article is the imperative need to rule out CDI in cancer patients admitted with diarrhea, given the high risk of mortality. In anticipation of a novel pandemic outbreak, it is advisable to enhance the judicious utilization of antibacterial agents while concurrently intensifying hand hygiene protocols. It is imperative to recognize the persistent prevalence of patients afflicted with CDI and accordingly ensure a steady allocation of beds for their management. By integrating lessons learned from the pandemic experience, we can strive towards optimizing patient outcomes and healthcare delivery in the post-COVID era.

## Figures and Tables

**Table 1:** Incidence rate of CDI and COVID-19 by total stays and admitted patients.

Variable	2020	2021	2022	*p* Value ^1^	*p* Value ^2^	*p* Value ^3^
Total stays of adult patients	80,611	87,167	92,594	-	-	-
Total admissions of adult patients	11,299	12,158	14,391	-	-	-
Total number of CDI patients	75	65	69	-	-	-
Total admissions of COVID-19 patients	829	867	841	-	-	-
Rate of CDI/10,000 stays	9.30	7.46	7.45	0.190	0.180	0.993
Rate of CDI/1000 admissions	6.64	5.35	4.79	0.199	0.049	0.527
Rate of COVID-19/10,000 stays	102.84	99.46	90.83	0.489	0.010	0.059
Rate of COVID-19/1000 admissions	73.37	71.31	58.44	0.543	<0.001	<0.001

^1^ Comparing 2020 and 2021; ^2^ Comparing 2020 and 2022; ^3^ Comparing 2021 and 2022; The infection rates were determined by dividing the number of cases by the total number of stays or admissions and multiplying by 10,000 in the first case and by 1000 in the second. CDI: *Clostridioides difficile* infection. Statistical significance was calculated by the χ^2^ test.

**Table 2:** Patient clinical and demographic characteristics.

Variable	2020 *n* = 75	2021 *n* = 65	2022 *n* = 69	*p* Value ^1^	*p* Value ^2^	*p* Value ^3^
Age, years	65.6 (16.4)	64.6 (17.4)	64.3 (21.4)	0.704	0.922	0.762
Male sex	39 (52.0)	22 (33.8)	33 (47.8)	0.031	0.617	0.100
**Department of admission**						
Internal Medicine	16 (21.3)	15 (23.1)	13 (18.8)	0.804	0.709	0.546
Emergency	32 (42.7)	23 (35.4)	30 (43.5)	0.378	0.921	0.220
Surgery	10 (13.3)	8 (12.3)	4 (5.8)	0.856	0.127	0.187
Intensive Care Unit	5 (6.7)	5 (7.7)	4 (5.8)	0.814	0.829	0.661
Oncology	6 (8.0)	6 (9.2)	15 (21.7)	0.795	0.019	0.046
Outpatient clinics	3 (4.0)	6 (9.2)	2 (2.9)	0.208	0.718	0.222
Other	3 (4.0)	2 (3.1)	1 (1.4)	0.769	0.352	0.524
**CDI origin**						
Nosocomial	24 (32.0)	19 (29.2)	22 (31.9)	0.723	0.988	0.739
Associated with health system	12 (16.0)	9 (13.8)	11 (15.9)	0.721	0.992	0.733
Community-acquired	39 (52.0)	37 (56.9)	36 (52.2)	0.559	0.983	0.581
**Days of admission in ward**						
Total days	12.7 (14.2)	22.0 (33.4)	13.9 (17.6)	0.458	0.594	0.265
Days pre-CDI	5.1 (7.7)	6.5 (11.0)	4.5 (9.3)	0.513	0.313	0.104
Days post-CDI	7.6 (11.5)	15.6 (27.3)	9.4 (12.0)	0.250	0.797	0.437
**Comorbidities**						
COVID-19	6 (8.0)	8 (12.3)	3 (4.3)	0.397	0.366	0.093
Diabetes mellitus	26 (34.7)	18 (27.7)	18 (26.1)	0.375	0.264	0.834
Chronic kidney disease	19 (25.3)	11 (16.9)	18 (26.1)	0.226	0.918	0.198
Chronic lung disease	11 (14.7)	5 (7.7)	3 (4.3)	0.196	0.037	0.414
Intestinal bowel disease	3 (4.0)	4 (6.2)	1 (1.4)	0.560	0.352	0.151
Gastric disease	24 (32.0)	17 (26.2)	25 (36.2)	0.448	0.592	0.209
Rheumatic disease	2 (2.7)	8 (12.3)	8 (11.6)	0.027	0.303	0.899
Cancer	19 (25.3)	12 (18.5)	21 (30.4)	0.606	0.404	0.110
**Cancer type**						
Lung	1 (1.3)	1 (1.5)	2 (2.9)			
Breast	2 (2.7)	1 (1.5)	3 (4.3)			
Gastric	1 (1.3)	1 (1.5)	0 (0.0)			
Colorectal	3 (4.0)	3 (4.6)	3 (4.3)			
Kidney	0 (0.0)	0 (0.0)	1 (1.4)			
Bladder	1 (1.3)	0 (0.0)	0 (0.0)	0.553	0.749	0.743
Gynecologic	4 (5.3)	1 (1.5)	3 (4.3)			
Blood	3 (4.0)	2 (3.1)	2 (2.9)			
Pancreas	0 (0.0)	2 (3.1)	3 (4.3)			
Bile ducts	0 (0.0)	1 (1.5)	1 (1.4)			
Liver	1 (1.3)	0 (0.0)	1 (1.4)			
Other	3 (4.0)	0 (0.0)	2 (2.9)			
**Cancer extension**						
Localized	4 (5.3)	3 (4.7)	8 (11.6)			
Metastasic	12 (16.0)	7 (10.8)	10 (14.5)	0.793	0.586	0.417
Unknown	3 (4.0)	2 (3.1)	2 (2.9)			
**Cancer therapy**						
Chemotherapy	9 (12.0)	6 (9.2)	12 (17.4)	0.407	0.573	0.338
Immunotherapy	5 (6.7)	2 (3.1)	5 (7.2)	0.552	0.848	0.337
Radiation therapy	1 (1.3)	0 (0.0)	3 (4.3)	0.644	0.165	0.069
**Treatments**						
Immunosuppressive treatment	17 (22.7)	12 (18.5)	20 (29.0)	0.540	0.386	0.153
Previous GI surgery	21 (28.0)	9 (13.8)	1 (1.4)	0.042	<0.001	0.006
Colonoscopy	0 (0.0)	6 (9.2)	2 (2.9)	0.007	0.138	0.122
Gastroscopy	5 (6.7)	12 (18.5)	8 (11.6)	0.033	0.303	0.265
H3PCDI	19 (25.3)	21 (32.3)	27 (39.1)	0.446	0.143	0.410
H6PCDI	21 (28.0)	21 (32.3)	30 (43.5)	0.615	0.060	0.183
AB3PCDI	47 (62.7)	42 (64.6)	37 (53.6)	0.811	0.271	0.196
PPI	64 (85.3)	35 (53.8)	34 (49.3)	<0.001	<0.001	0.564
**Charlson index**						
Index > 5	32 (42.7)	16 (24.6)	28 (40.6)	0.024	0.799	0.049
**McCabe score**						
Rapidly fatal disease	15 (20.0)	13 (20.0)	19 (27.5)	1.000	0.287	0.306
Ultimately fatal disease	23 (30.7)	16 (24.6)	14 (20.3)	0.425	0.154	0.548
Non-fatal disease	37 (49.3)	36 (55.4)	35 (50.7)	0.474	0.867	0.589
**Laboratory analyses**						
Leukocytes, ×10^9^/L	10,736 (5711)	11,091 (7875)	13,947 (12,966)	0.644	0.193	0.125
Albumin, g/dL	3.3 (0.8)	3.2 (1.0)	3.2 (0.7)	0.526	0.499	0.666
C-reactive protein, mg/L	9.8 (10.3)	8.2 (10.3)	9.4 (8.8)	0.230	0.645	0.064
**Outcomes**						
Recurrences	8 (10.7)	7 (10.8)	5 (7.2)	0.984	0.474	0.475
Deceased	10 (13.3)	6 (9.2)	11 (15.9)	0.447	0.658	0.243

^1^ Comparing 2020 and 2021; ^2^ Comparing 2020 and 2022; ^3^ Comparing 2021 and 2022; Days pre-CDI is the number of days of admission before *Clostridioides difficile* diagnosis; Days post-CDI is the number of days of admission after *Clostridioides difficile* diagnosis; AB3PCDI: Antibiotics 3 months prior to CDI; CDI: *Clostridioides difficile* infection; GI: Gastrointestinal; H3PCDI: Hospitalization 3 months prior to CDI; H6PCDI: Hospitalization 6 months prior to CDI; PPI: Proton pump inhibitors. The results of qualitative variables are shown as numbers and percentages, and statistical significance was calculated by the χ^2^ test. The results of quantitative variables are shown as medians and interquartile ranges, and statistical significance was calculated by the Mann–Whitney U test. Statistical analysis of cancer types has been performed globally due to the low number of cases of each individual cancer.

**Table 3:** Risk factors for mortality of patients with CDI.

Variable	Survivors *n* = 182	Deceased *n* = 27	*p* Value
Age, years	63.9 (18.8)	71.2 (13.9)	0.054
Male sex	80 (44.0)	14 (51.9)	0.442
**Department of admission**			
Internal Medicine	38 (20.9)	6 (22.2)	0.873
Emergency	79 (43.4)	6 (22.2)	0.036
Surgery	22 (12.1)	0 (0.0)	0.056
Intensive Care	11 (6.0)	3 (11.1)	0.325
Oncology	16 (8.8)	11 (40.7)	<0.001
Outpatient clinics	11 (6.0)	0 (0.0)	
Other	5 (2.7)	1 (3.7)	0.781
**CDI origin**			
Nosocomial	53 (29.1)	12 (44.4)	0.108
Associated with health system	28 (15.4)	4 (14.8)	0.938
Community-acquired	101 (55.5)	11 (40.7)	0.151
**Days of admission in ward**			
Total days	15.8 (24.4)	17.4 (9.9)	0.740
Days pre-CDI	5.1 (9.5)	6.9 (8.0)	0.367
Days post-CDI	10.7 (19.3)	10.6 (9.5)	0.984
**Comorbidities**			
COVID-19	14 (7.7)	3 (11.1)	0.544
Diabetes mellitus	53 (29.1)	9 (33.3)	0.655
Chronic kidney disease	34 (1.6)	14 (51.9)	<0.001
Chronic lung disease	15 (8.2)	4 (14.8)	0.268
Intestinal bowel disease	8 (4.4)	0 (0.0)	0.267
Gastric disease	56 (30.8)	10 (37.0)	0.513
Rheumatic disease	16 (8.8)	2 (7.4)	0.811
Cancer	31 (17.0)	11 (40.7)	0.007
**Cancer type**			
Lung	1 (0.5)	3 (11.1)	
Breast	5 (2.7)	1 (3.7)	
Gastric	2 (1.1)	0 (0.0)	
Colorectal	8 (4.4)	1 (3.7)	
Kidney	0 (0.0)	1 (3.7)	
Bladder	0 (0.0)	1 (3.7)	<0.001
Gynecologic	7 (3.8)	1 (3.7)	
Blood	6 (3.3)	1 (3.7)	
Pancreas	5 (2.7)	0 (0.0)	
Bile ducts	0 (0.0)	2 (7.4)	
Liver	2 (1.1)	0 (0.0)	
Other	2 (1.1)	3 (11.1)	
**Cancer extension**			
Localized	13 (7.1)	2 (7.4)	
Metastasic	18 (9.9)	11 (40.7)	<0.001
Unknown	6 (3.3)	1 (3.7)	
**Cancer therapy**			
Chemotherapy	20 (11.0)	7 (25.9)	0.084
Immunotherapy	9 (4.9)	3 (11.1)	0.414
Radiation therapy	3 (1.6)	1 (3.7)	0.551
**Treatments**			
Immunosuppressive treatment	36 (19.8)	13 (48.1)	0.001
Previous GI surgery	28 (15.4)	3 (11.1)	0.560
Colonoscopy	4 (2.2)	4 (14.8)	0.001
Gastroscopy	20 (11.0)	5 (18.5)	0.261
H3PCDI	52 (28.6)	15 (55.5)	<0.001
H6PCDI	57 (31.3)	15 (55.5)	0.008
AB3PCDI	107 (58.8)	19 (70.4)	0.251
PPI	111 (61.0)	22 (81.4)	0.116
**Charlson index**			
Index > 5	58 (31.9)	18 (66.7)	<0.001
**McCabe score**			
Rapidly fatal disease	24 (13.2)	23 (85.2)	
Ultimately fatal disease	50 (27.5)	3 (11.1)	<0.001
Non-fatal disease	107 (58.8)	1 (3.7)	
**Laboratory analyses**			
Leukocytes	11,299 (6906)	15912 (18,580)	0.016
Albumin	3.3 (0.8)	2.9 (0.6)	0.017
C-reactive protein	8.1 (8.8)	15.9 (12.9)	<0.001
**Outcomes**			
Recurrences	18 (9.9)	2 (7.4)	0.682

Days pre-CDI is the number of days of admission before *Clostridioides difficile* diagnosis; Days post-CDI is the number of days of admission after *Clostridioides difficile* diagnosis; AB3PCDI: Antibiotics 3 months prior to CDI; CDI: *Clostridioides difficile* infection; GI: Gastrointestinal; H3PCDI: Hospitalization 3 months prior to CDI; H6PCDI: Hospitalization 6 months prior to CDI; PPI: Proton pump inhibitors. The results of qualitative variables are shown as numbers and percentages, and statistical significance was calculated by the χ^2^ test. The results of quantitative variables are shown as medians and interquartile ranges, and statistical significance was calculated by the Mann–Whitney U test. Statistical analysis of cancer types has been performed globally due to the low number of cases of each individual cancer.

**Table 4:** Risk factors for prolonged hospital stays of patients with CDI.

Variable	Days Post-CDI ≤ 5 *n* = 111	Days Post-CDI > 5 *n* = 98	*p* Value
Age, years	61.9 (18.6)	68.34 (17.6)	0.012
Male sex	47 (42.3)	47 (48.0)	0.415
**Department of admission**			
Internal Medicine	18 (16.2)	26 (26.5)	0.186
Emergency	57 (51.4)	28 (28.6)	<0.001
Surgery	9 (8.1)	13 (13.3)	0.225
Intensive Care	5 (4.5)	9 (9.2)	0.133
Oncology	9 (8.1)	18 (18.4)	0.027
Other	2 (1.8)	4 (4.1)	0.324
**CDI origin**			
Nosocomial	24 (21.6)	41 (41.8)	0.158
Associated with health system	13 (11.7)	19 (19.4)	0.124
Community-acquired	14 (12.6)	38 (38.8)	<0.001
**Days of admission in ward**			
Total days	4.7 (7.0)	28.7 (27.9)	<0.001
Days pre-CDI	3.0 (6.3)	8.0 (11.3)	<0.001
Days post-CDI	1.7 (1.9)	20.9 (22.7)	<0.001
**Comorbidities**			
COVID-19	8 (7.2)	9 (9.2)	0.602
Diabetes mellitus	24 (21.6)	38 (38.8)	0.007
Chronic kidney disease	20 (18.0)	28 (28.6)	0.070
Chronic lung disease	9 (8.1)	10 (10.2)	0.599
Intestinal bowel disease	5 (4.5)	3 (3.1)	0.587
Gastric disease	33 (29.7)	33 (33.7)	0.540
Rheumatic disease	7 (6.3)	11 (11.2)	0.206
Cancer	21 (18.9)	31 (31.6)	0.050
**Cancer type**			
Lung	1 (0.9)	3 (3.1)	
Breast	3 (2.7)	3 (3.1)	
Gastric	0 (0.0)	2 (2.0)	
Colorectal	4 (3.6)	5 (5.1)	
Kidney	0 (0.0)	1 (1.0)	
Bladder	1 (0.9)	0 (0.0)	
Gynecologic	4 (3.6)	4 (4.1)	0.241
Blood	5 (4.5)	2 (2.0)	
Pancreas	2 (1.8)	3 (3.1)	
Bile ducts	0 (0.0)	2 (2.0)	
Liver	0 (0.0)	2 (2.0)	
Other	1 (0.9)	4 (4.1)	
**Cancer extension**			
Localized	3 (2.7)	12 (12.2)	0.026
Metastasic	13 (11.7)	16 (16.3)	
Unknown	5 (4.5)	2 (2.0)	
**Cancer therapy**			
Chemotherapy	11 (9.9)	16 (16.3)	0.301
Immunotherapy	9 (8.1)	3 (3.1)	0.223
Radiation therapy	0 (0.0)	4 (4.1)	0.008
**Treatments**			
Immunosupressive treatment	19 (17.1)	30 (30.6)	0.022
Previous GI surgery	13 (11.7)	18 (18.4)	0.177
Colonoscopy	6 (5.4)	2 (2.0)	0.206
Gastroscopy	9 (8.1)	16 (16.3)	0.068
H3PCDI	27 (24.3)	40 (40.8)	0.019
H6PCDI	30 (27.0)	42 (42.9)	0.014
AB3PCDI	59 (53.2)	67 (68.4)	0.025
PPI	65 (58.6)	68 (69.4)	0.195
**Charlson index**			
Index > 5	29 (26.1)	47 (48.0)	0.001
**McCabe score**			
Rapidly fatal disease	19 (17.1)	28 (28.6)	
Ultimately fatal disease	19 (17.1)	34 (34.7)	<0.001
Non-fatal disease	72 (64.9)	36 (36.7)	
Laboratory analyses			
Leukocytes	11,688 (10,109)	12,141 (8454)	0.729
Albumin	3.6 (0.8)	2.9 (0.7)	<0.001
C-reactive protein	6.8 (9.4)	11.8 (9.6)	<0.001
**Outcomes**			
Recurrences	10 (9.0)	10 (10.2)	0.769
Deceased	10 (9.0)	17 (17.3)	0.073

Days post-CDI is the number of days of admission after Clostridioides difficile diagnosis; AB3PCDI: Antibiotics 3 months prior to CDI; CDI: Clostridioides difficile infection; GI: Gastrointestinal; H3PCDI: Hospitalization 3 months prior to CDI; H6PCDI: Hospitalization 6 months prior to CDI; PPI: Proton pump inhibitors. The results of qualitative variables are shown as numbers and percentages, and statistical significance was calculated by the χ^2^ test. The results of quantitative variables are shown as medians and interquartile ranges, and statistical significance was calculated by the Mann–Whitney U test. Statistical analysis of cancer types has been performed globally due to the low number of cases of each individual cancer.

## Data Availability

Dataset available on request from the authors.

## References

[1] Magill S.S., O’Leary E., Janelle S.J., Thompson D.L., Dumyati G., Nadle J., Wilson L.E., Kainer M.A., Lynfield R., Greissman S. (2018). Changes in prevalence of health care-associated infections in U.S. Hospitals. N. Engl. J. Med..

[2] Czepiel J., Dróżdż M., Pituch H., Kuijper E.J., Perucki W., Mielimonka A., Goldman S., Wultańska D., Garlicki A., Biesiada G. (2019). *Clostridium difficile* infection: Review. Eur. J. Clin. Microbiol. Infect. Dis..

[3] Sayedy L., Kothari D., Richards R.J. (2010). Toxic megacolon associated *Clostridium difficile* colitis. World J. Gastrointest. Endosc..

[4] Guh A.Y., Mu Y., Winston L.G., Johnston H., Olson D., Farley M.M., Wilson L.E., Holzbauer S.M., Phipps E.C., Dumyati G.K. (2020). Trends in U.S. burden of *Clostridioides difficile* infection and outcomes. N. Engl. J. Med..

[5] Bouza E., Cobo J., Rodríguez-Hernández M.J., Salavert M., Horcajada J.P., Iribarren J.A., Obi E., Lozano V., Maratia S., Cuesta M. (2021). Economic burden of recurrent *Clostridioides difficile* infection in adults admitted to Spanish hospitals. A multicentre retrospective observational study. Rev. Esp. Quimioter..

[6] Olona M., Limón E., Barcenilla F., Grau S., Gudiol F. (2012). Prevalence of nosocomial infections in acute care hospitals in Catalonia (VINCat Program). Enferm. Infecc. Microbiol. Clin..

[7] Sopena N., Freixas N., Bella F., Pérez J., Hornero A., Limon E., Gudiol F., Pujol M. (2019). Impact of a training program on the surveillance of *Clostridioides difficile* infection. Epidemiol. Infect..

[8] Leffler D.A., Lamont J.T. (2015). *Clostridium difficile* infection. N. Engl. J. Med..

[9] McDonald L.C., Gerding D.N., Johnson S., Bakken J.S., Carroll K.C., Coffin S.E., Dubberke E.R., Garey K.W., Gould C.V., Kelly C. (2018). Clinical practice guidelines for *Clostridium difficile* infection in adults and children: 2017 update by the Infectious Diseases Society of America (IDSA) and Society for Healthcare Epidemiology of America (SHEA). Clin. Infect. Dis..

[10] De Roo A.C., Regenbogen S.E. (2020). *Clostridium difficile* infection: An epidemiology update. Clin. Colon Rectal. Surg..

[11] Di Bella S., Sanson G., Monticelli J., Zerbato V., Principe L., Giuffrè M., Pipitone G., Luzzati R. (2024). *Clostridioides difficile* infection: History, epidemiology, risk factors, prevention, clinical manifestations, treatment, and future options. Clin. Microbiol. Rev..

[12] Fu Y., Luo Y., Grinspan A.M. (2021). Epidemiology of community-acquired and recurrent *Clostridioides difficile* infection. Therap. Adv. Gastroenterol..

[13] Luo Y., Grinspan L.T., Fu Y., Adams-Sommer V., Willey D.K., Patel G., Grinspan A.M. (2021). Hospital-onset *Clostridioides difficile* infections during the COVID-19 pandemic. Infect. Control Hosp. Epidemiol..

[14] Wee L.E.I., Conceicao E.P., Tan J.Y., Magesparan K.D., Amin I.B.M., Ismail B.B.S., Toh H.X., Jin P., Zhang J., Wee E.G.L. (2021). Unintended consequences of infection prevention and control measures during COVID-19 pandemic. Am. J. Infect. Control.

[15] Gerhards C., Steingass M., Heininger A., Lange B., Hetjens M., Gerigk M., Neumaier M., Evliyaoglu O., Kittel M. (2024). The impact of clinical factors and SARS-CoV-2 variants on antibody production in vaccinated German healthcare professionals infected either with the delta or the omicron variant. Vaccines.

[16] Ponce-Alonso M., Sáez de la Fuente J., Rincón-Carlavilla A., Moreno-Nunez P., Martínez-García L., Escudero-Sánchez R., Pintor R., García-Fernández S., Cobo J. (2021). Impact of the coronavirus disease 2019 (COVID-19) pandemic on nosocomial *Clostridioides difficile* infection. Infect. Control Hosp. Epidemiol..

[17] Hazel K., Skally M., Glynn E., Foley M., Burns K., O’Toole A., Boland K., Fitzpatrick F. (2022). The other ‘C’: Hospital-acquired *Clostridioides difficile* infection during the coronavirus disease 2019 (COVID-19) pandemic. Infect. Control Hosp. Epidemiol..

[18] Bentivegna E., Alessio G., Spuntarelli V., Luciani M., Santino I., Simmaco M., Martelletti P. (2021). Impact of COVID-19 prevention measures on risk of health care-associated *Clostridium difficile* infection. Am. J. Infect. Control.

[19] Ochoa-Hein E., Rajme-López S., Rodríguez-Aldama J.C., Huertas-Jiménez M.A., Chávez-Ríos A.R., de Paz-García R., Haro-Osnaya A., González-Colín K.K., González-González R., González-Lara M.F. (2021). Substantial reduction of healthcare facility-onset *Clostridioides difficile* infection (HO-CDI) rates after conversion of a hospital for exclusive treatment of COVID-19 patients. Am. J. Infect. Control.

[20] Iftimie S., López-Azcona A.F., Vallverdú I., Hernández-Flix S., de Febrer G., Parra S., Hernández-Aguilera A., Riu F., Joven J., Andreychuk N. (2021). First and second waves of coronavirus disease-19: A comparative study in hospitalized patients in Reus, Spain. PLoS ONE.

[21] Iftimie S., López-Azcona A.F., Lozano-Olmo M.J., Hernández-Aguilera A., Sarrà-Moretó S., Joven J., Camps J., Castro A. (2022). Characteristics of hospitalized patients with SARS-CoV-2 infection during successive waves of the COVID-19 pandemic in a reference hospital in Spain. Sci. Rep..

[22] Iftimie S., López-Azcona A.F., Lozano-Olmo M.J., Naval-Ferrando À., Domingo-Cortés V., Castañé H., Jiménez-Franco A., Hernández-Aguilera A., Guilarte C., Riu F. (2023). Retrospective analysis of vaccination status and predominant viral variants in patients hospitalized with COVID-19 in Reus, Spain. Viruses.

[23] (2019). Manual VINCat. https://catsalut.gencat.cat/web/.content/minisite/vincat/documents/manuals/Manual-VINCat-2019.pdf.

[24] Kreger B.E., Craven D.E., Carling P.C., McCabe W.R. (1980). Gram-negative bacteremia. III. Reassessment of etiology, epidemiology and ecology in 612 patients. Am. J. Med..

[25] Berkman L.F., Leo-Summers L., Horwitz R.I. (1992). Emotional support and survival after myocardial infarction. A prospective, population-based study of the elderly. Ann. Intern. Med..

[26] Crobach M.J., Planche T., Eckert C., Barbut F., Terveer E.M., Dekkers O.M., Wilcox M.H., Kuijper E.J. (2016). European Society of Clinical Microbiology and Infectious Diseases: Update of the diagnostic guidance document for *Clostridium difficile* infection. Clin. Microbiol. Infect..

[27] Iftimie S., López-Azcona A.F., Vicente-Miralles M., Descarrega-Reina R., Hernández-Aguilera A., Riu F., Simó J.M., Garrido P., Joven J., Camps J. (2020). Risk factors associated with mortality in hospitalized patients with SARS-CoV-2 infection. A prospective, longitudinal, unicenter study in Reus, Spain. PLoS ONE.

[28] Lang K., Atchison T.J., Singh P., Kline D.M., Odei J.B., Martin J.L., Smyer J.F., Day S.R., Hebert C.L. (2023). Describing the monthly variability of hospital-onset *Clostridioides difficile* during early coronavirus disease 2019 (COVID-19) using electronic health record data. Infect. Control Hosp. Epidemiol..

[29] Suarez L., Kim J., Freedberg D.E., Lebwohl B. (2022). Risk of healthcare-associated *Clostridioides difficile* infection during pandemic preparation: A retrospective cohort study. Gastro. Hep. Adv..

[30] Wright L.M., Skinner A.M., Cheknis A., McBurney C., Ge L., Pacheco S.M., Leehey D., Gerding D.N., Johnson S. (2023). Effect of the COVID-19 pandemic on rates and epidemiology of *Clostridioides difficile* infection in one VA hospital. Antibiotics.

[31] Allegretti J.R., Nije C., McClure E., Redd W.D., Wong D., Zhou J.C., Bazarbashi A.N., McCarty T.R., Hathorn K.E., Shen L. (2021). Prevalence and impact of *Clostridioides difficile* infection among hospitalized patients with coranavirus disease 2019. JGH Open.

[32] Sinnathamby E.S., Mason J.W., Flanagan C.J., Pearl N.Z., Burroughs C.R., De Witt A.J., Wenger D.M., Klapper V.G., Ahmadzadeh S., Varrassi G. (2023). *Clostridioides difficile* infection: A clinical review of pathogenesis, clinical considerations, and treatment strategies. Cureus.

[33] Tossens B., Barthelme P., Briquet C., Belkhir L., Ngyuvula E., Soumillion K., Verroken A., Rodriguez-Villalobos H., Delmée M., Anantharajah A. (2023). Impact of the COVID-19 pandemic on *Clostridioides difficile* infection in a tertiary healthcare institution in Belgium. Acta Clin..

[34] Wilson Dib R., Spallone A., Khawaja F., Feldman A., Cantu S., Chemaly R.F. (2023). The impact of the COVID-19 pandemic on hospital-acquired infections at a comprehensive cancer center. Am. J. Infect. Control.

[35] Deac I.Ş., Ofrim A.M., Fărcaş R.A., Grad S., Popa Ş.L., Dumitraşcu D.L. (2024). The management of *Clostridioides difficile* infection: From empirism to evidence. Med. Pharm. Rep..

[36] Abubakar U., Awaisu A., Khan A.H., Alam K. (2023). Impact of COVID-19 pandemic on healthcare-associated infections: A systematic review and meta-analysis. Antibiotics.

[37] Hartnett K.P., Kite-Powell A., DeVies J., Coletta M.A., Boehmer T.K., Adjemian J., Gundlapalli A.V. (2020). Impact of the COVID-19 pandemic on emergency department visits—United States, January 1, 2019-May 30, 2020. MMWR Morb. Mortal. Wkly. Rep..

[38] McCracken C.E., Gander J.C., McDonald B., Goodrich G.K., Tavel H.M., Basra S., Weinfield N.S., Ritzwoller D.P., Roblin D.W., Davis T.L. (2023). Impact of COVID-19 on trends in outpatient clinic utilization: A tale of 2 departments. Med. Care.

[39] O’Reilly-Shah V.N., Van Cleve W., Long D.R., Moll V., Evans F.M., Sunshine J.E., Kassebaum N.J., Harrison E.M., Jabaley C.S. (2020). Impact of COVID-19 response on global surgical volumes: An ongoing observational study. Bull. World Health Organ..

[40] Gluckman T.J., Wilson M.A., Chiu S.T., Penny B.W., Chepuri V.B., Waggoner J.W., Spinelli K.J. (2020). Case rates, treatment approaches, and outcomes in acute myocardial infarction during the coronavirus disease 2019 pandemic. JAMA Cardiol..

[41] Alquézar-Arbé A., Miró Ò., Piñera P., Jacob J., Martín A., Agra Montava I., Llorens P., Jiménez S., Burillo-Putze G., García-Lamberechts E.J. (2021). Analysis of the evolution of patients attended in Spanish emergency departments during the first wave of the pandemic. An. Sist. Sanit. Navar..

[42] World Health Organization Promoting Antimicrobial Stewardship to Tackle Antimicrobial Resistance. https://www.who.int/europe/activities/promoting-antimicrobial-stewardship-to-tackle-antimicrobial-resistance.

[43] Vintila B.I., Arseniu A.M., Morgovan C., Butuca A., Bîrluțiu V., Dobrea C.M., Rus L.L., Ghibu S., Bereanu A., Arseniu R. (2024). A real-world study on the clinical characteristics, outcomes, and relationship between antibiotic exposure and *Clostridioides difficile* infection. Antibiotics.

[44] Solomon K., Martin A.J., O’Donoghue C., Chen X., Fenelon L., Fanning S., Kelly C.P., Kyne L. (2013). Mortality in patients with *Clostridium difficile* infection correlates with host pro-inflammatory and humoral immune responses. J. Med. Microbiol..

[45] Wilson V., Cheek L., Satta G., Walker-Bone K., Cubbon M., Citron D., Gerding D.N., Llewelyn M.J. (2010). Predictors of death after *Clostridium difficile* infection: A report on 128 strain-typed cases from a teaching hospital in the United Kingdom. Clin. Infect. Dis..

[46] Kassam Z., Cribb Fabersunne C., Smith M.B., Alm E.J., Kaplan G.G., Nguyen G.C., Ananthakrishnan A.N. (2016). *Clostridium difficile* associated risk of death score (CARDS): A novel severity score to predict mortality among hospitalised patients with *C. difficile* infection. Aliment. Pharmacol. Ther..

[47] Lee H.Y., Hsiao H.L., Chia C.Y., Cheng C.W., Tsai T.C., Deng S.T., Chen C.L., Chiu C.H. (2019). Risk factors and outcomes of *Clostridium difficile* infection in hospitalized patients. Biomed. J..

